# Word order and context in sentence processing: evidence from L1 and L2 Russian

**DOI:** 10.3389/fpsyg.2024.1344366

**Published:** 2024-03-20

**Authors:** Natalia Slioussar, Maria Harchevnik

**Affiliations:** ^1^School of Linguistics, National Research University Higher School of Economics, Moscow, Russia; ^2^Institute for Cognitive Studies, Saint Petersburg State University, Saint Petersburg, Russia

**Keywords:** word order, context, information structure, second language processing, Russian

## Abstract

**Introduction:**

In this paper, we studied how native (L1) speakers of Russian and speakers of Mandarin Chinese learning Russian as a foreign language (L2) process Russian sentences with different word orders. We compared SVO (canonical) and OVS (non-canonical) orders in isolation and in context. Experiments focusing on the L2 processing of different word orders are still not very numerous, and those using context are extremely rare.

**Methods:**

In Experiment 1, target sentences were presented in isolation. In Experiment 2, one-sentence contexts introduced one NP mentioned in the target sentence, either the first (so that given information preceded new information, which is characteristic for Russian and many other languages) or the second. As a result, two factors could be compared: the syntactic (word order) and the contextual (whether the context is appropriate from the information-structural perspective). We used different measures to capture online and offline effects: word-by-word reading times, question-answering accuracy and sentence rating on a 1 to 5 scale (for L1 participants).

**Results and discussion:**

In both experiments, RTs and question-answering accuracy data showed that non-canonical orders were difficult for L2 participants, but not for L1 participants. However, L1 participants gave non-canonical orders lower ratings in isolation, presumably because in naturally occurring texts, they are used only in particular contexts. As for the context factor in Experiment 2, some effects were the same for L1 and L2 processing: all participants read given NPs faster than new ones and preferred sentences with a ‘given – new’ word order. The latter may reflect the universal principles of narrative coherence. However, unlike native speakers, L2 readers are not sensitive to more subtle contextual requirements of different word orders.

## 1 Introduction

In most languages, different word orders are possible in a sentence, although such alternations are more diverse and more widespread in some languages than in the others. They are primarily associated with the information structure of the sentence (which information is new or given, salient or backgrounded). One word order (the most frequent, with the least specific information-structural requirements) is termed canonical or basic. Many studies are dedicated to processing sentences with different word orders, both by native (L1) speakers and by second language (L2) learners, for whom acquiring the rules underlying word order alternations was shown to be particularly difficult in various languages.

In the first processing experiments with L1 readers, different orders were presented in isolation. But, since their use depends on information structure, subsequent studies presented them in contexts. L2 processing experiments are still not very numerous, and those using contexts are extremely rare. However, it would be interesting to find out whether L2 readers are sensitive to various contextual requirements of different orders, and how they differ from L1 readers in this respect.

In the present study, we aimed to fill this gap. We compared how native speakers of Russian and speakers of Mandarin Chinese learning Russian as a foreign language process Russian sentences with different word orders. In Experiment 1, these sentences were presented in isolation, while in Experiment 2, we used one-sentence contexts satisfying or violating information-structural requirements of these sentences. We used different measures (word-by-word reading times, question-answering accuracy and sentence rating on a 1–5 scale) to capture online and offline effects.

The structure of the paper is as follows. In the next section, we briefly introduce the main properties of word order in Russian and in Chinese. Then we give an overview of L1 and L2 processing studies focusing on word order alternations. After that, we turn to the present study.

### 1.1 Word order in Russian and in Mandarin Chinese

We selected sentences with a subject NP, an object NP and a transitive verb for our study. Both in Russian and in Mandarin Chinese (Putonghua), the basic word order is SVO (subject – verb – object) in such sentences (Dryer, 2005).^1^ Both languages allow for certain word order alternations, mainly triggered by the information structure. Both languages, especially in written texts, prefer to put given information before new information, when it is possible to change the word order in the sentence accordingly. However, the two languages are very different in other respects: while Russian is a morphologically rich inflected language with morphological case marking, Chinese is an isolating language: most words consist of a single morpheme and have no inflectional morphology. Russian allows for more diverse word order alternations, and in general, possible word orders in these languages are not similar to each other.

In a Russian sentence with a subject, an object and a verb, all six computationally possible orders are attested. Russian has six cases, and does not use prepositions not only with direct objects, but often also with various indirect ones. Due to morphological case marking, subjects, direct and indirect objects can usually be told apart unambiguously.^2^ Slioussar and Makarchuk (2022) conducted a corpus study showing the prevalence of these six orders in more formal and less formal written and oral texts. The basic SVO order clearly prevails everywhere. The second most frequent order in narrative written texts is OVS, and this was one of the reasons to choose it for our experiments.

Word order alternations in Russian and information-structural requirements associated with them were studied by many authors working in different frameworks (e.g., Sirotinina, 1965; Kovtunova, 1976; Krylova, 1992; Bailyn, 1995; Yanko, 2001; Slioussar, 2007; Titov, 2017, 2020). For some orders, these requirements are easier to formulate, while for the others, they are a matter of debate [for example, Slioussar and Makarchuk (2022) discuss this problem for SOV orders]. OVS orders are relatively transparent in this respect, and this was the second reason to choose them. In the majority of cases, they are used when the subject is in focus (new information), while the object is topicalized (usually given).

Apart from information structure, the choice of word order may be affected by the argument prominence hierarchy: humans > animals > inanimates (Titov, 2017; Vihman and Nelson, 2019). Namely, when arguments have the same information-structural status (e.g., are both new), a non-canonical word order may still be used so that NPs denoting humans could precede NPs denoting animals and inanimate things. Since we wanted to focus on information structure in this study, we balanced arguments for animacy in our target sentences.

If the argument prominence hierarchy is controlled for, the canonical SVO order is the only one that is fully appropriate to use in isolation. The widest range of contexts is associated with it. In particular, when the subject is new and the object is given, Russian speakers can use not only OVS, but also SVO, shifting the main stress on the subject, as in (1)–(2) (the NP bearing the main stress is underlined). However, this is more characteristic for dialogues than for narrative texts (Kodzasov, 1996).

(1) *Kto videl Petju?*

who_NOM.SG_ saw Petya_ACC.SG_.

‘Who saw Petya?’

(2) а. *Petju videl Vasja* (OVS)

Petya_ACC.SG_ saw Vasya_NOM.SG_.

b. *Vasja videl Petju* (SVO)

Vasya_NOM.SG_ saw Petya_ACC.SG_.

‘Vasya saw Petya.’

There were also other reasons to include the OVS order in our experiments. As we show below, such orders are virtually unattested in Chinese, which creates a challenge for Chinese learners of Russian. In addition to that, we wanted to have sentences with an inverted order of arguments — figuring out the predicate-argument structure appears to be the main problem associated with processing of non-canonical orders. Thus, we had SVO and OVS sentences in our study.

Mandarin Chinese (Putonghua) is an isolating language. Therefore, while Russian can rely on case marking to tell arguments apart, word order and context play a crucial role for this in Chinese. Nevertheless, Chinese allows for certain word order alternations (Sun and Givón, 1985; Sun, 2006). In particular, SOV orders are very widespread, especially in spoken language in northern dialects (Li and Thompson, 1974; Gao, 2008). Sentences in which a non-subject NP precedes the verb, while the subject follows it are possible, but with various intransitive verbs (Gao, 2008): these NPs may denote location, time etc. Transitive verbs selecting direct objects are not used in OVS orders, which may create specific problems with Russian OVS sentences for Chinese L2 learners.

### 1.2 L1 processing of sentences with different word orders

Many studies on different languages focused on L1 processing of different word orders. In most of them, target sentences appeared in isolation (Frazier and Flores d’Arcais, 1989; Hyönä and Hujanen, 1997; Gibson, 1998; Bader and Meng, 1999; Clahsen and Featherston, 1999; Sekerina, 1999; Stojanovic, 1999; Miyamoto and Takahashi, 2000; Vasishth, 2002; Erdocia et al., 2009, among others). Non-canonical orders were found to be more difficult to process than canonical ones, although these differences did not reach significance in some studies — presumably, due to the fact that non-canonical orders may be very widespread in some languages (although they are still much less frequent than canonical ones).

However, non-canonical orders have contextual requirements and sound less natural in isolation. To find out to what extent processing difficulties may be associated with word order alternations *per se* and with context, several authors introduced context sentences in their experiments. Let us look at some of these studies in more detail.

Bornkessel et al. (2003) presented German sentences with different word orders in isolation and using questions as contexts. ERPs and word-by-word reading times were recorded. Non-canonical orders were more difficult than canonical ones in isolation. Context eliminated this difficulty, but only partially. Some signature effects associated with the syntactic and contextual factor were identified. These results were supported and extended in later studies (Schumacher and Hung, 2012; Burmester et al., 2014).

Kaiser and Trueswell (2004) examined Finnish sentences with SVO and OVS orders. Their syntactic and information-structural properties are similar to those in Russian, so this study is especially relevant for us. In the first experiment, Kaiser and Trueswell presented these orders after two-sentence contexts, as in (3a-b), and measured word-by-word reading times. The second context sentence introduced one NP from the target sentence, either the first one, as in (3c) (creating a ‘given – new’ order in the target sentence), or the second one, as in (3d) (creating a ‘new – given’ order in the target sentence, not characteristic for narrative texts in Finnish, like in Russian). As a result, they could compare the effects of the word order and context type factors. Both factors were significant: sentences in ‘new – given’ contexts and sentences with non-canonical word orders were read more slowly.

(3) a. *Lotta etsi eilen sieniä metsässä.*

‘Lotta looked for mushrooms yesterday in the forest.’

b. *Hän huomasi heinikossa jäniksen joka liikkui* var*ovasti eteenpäin.*

(s)he noticed grass_LOC_ hare_ACC_ that was-moving carefully forward.

c. *Jänistä seurasi hiiri ja linnut lauloivat.*

hare_PART_ followed mouse_NOM_ and birds were-singing.

d. *Hiiri seurasi jänistä ja linnut lauloivat.*

mouse_NOM_ followed hare_PART_ and birds were-singing.

In the second experiment, participants’ eye movements were tracked as they looked at stimulus pictures and listened to their descriptions (including context sentences and target SVO or OVS sentences). If the first NP in the target sentence referred to a given referent (that was mentioned in the preceding context), sentences with the OVS order demonstrated anticipatory eye movements toward the discursively new referent even before the participants received sufficient acoustic information to recognize the second NP. This was not the case for the SVO condition. This shows that Finnish speakers expect the OVS order to be used in certain contexts, while the contextual requirements of the canonical SVO order are much wider.

Sekerina (2003) was the first to compare Russian sentences with different word orders in isolation and in context. However, in her study, one-sentence contexts which did not vary across conditions. A general facilitative effect of context was reported, but non-canonical orders still had longer reading times than canonical ones.

Slioussar’s (2011a) study on Russian followed the same logic as the first experiment by Kaiser and Trueswell (2004): ‘given – new’ and ‘new – given’ contexts were used. However, Slioussar compared more complex word orders with three argument NPs (a subject, a direct and an indirect object) and had more complex contexts where two out of three NPs were introduced. The context factor was significant, while the word order factor was not: all orders were equally easy to read in an appropriate context. Having longer sentences, Slioussar also could describe in more detail how different contexts affect processing word-by-word.

In several studies on Spanish, Gattei et al. (2015, 2017, 2021) focused on another aspect of processing different word orders: on the problem of establishing predicate-argument structure. In Spanish, like in Russian (e.g., Slioussar, 2011b), agentive subjects tend to precede patientive objects (resulting in the prevalence of SVO orders with active verbs), but patientive subjects tend to follow experiencer objects (resulting in the prevalence of OVS orders in the relevant group of psych verbs).^3^ Gattei and colleagues demonstrated that these two groups of verbs have distinct processing patterns. In particular, Gattei et al. (2021) used the same two types of contexts, as Kaiser and Trueswell (2004) and Slioussar (2011a), but compared SVO and OVS orders in these two groups of verbs in an eye-tracking-while-reading study. Several diverse measures were used in the study (‘early’ and ‘late’ eye-movement measures, accuracy and response times to comprehension questions), and all three factors played a significant role at least for some of them.

### 1.3 L2 processing of sentences with different word orders

In this section, we will first discuss some general ideas that may be important for our study and then the experiment by Laleko (2022) that is especially relevant for us. Many authors, especially in formal approaches to second language acquisition, have noted that various phenomena at the interface between the grammar and information structure present a challenge even to advanced L2 learners (e.g., DeKeyser, 2005; Callies, 2009; Sorace, 2011). It is easier to master grammatical rules underlying various constructions than to grasp how these constructions are used depending on the discourse context. Sorace generalizes this insight in her Interface Hypothesis (Sorace and Serratrice, 2009; Sorace, 2011, 2012). She assumes that language processing is modular, so it can be expected that using the information within the computational system is easier than figuring out the interactions between the modules. Moreover, external interfaces (e.g., syntax interacting with discourse) are expected to be more challenging than internal ones (e.g., lexicon interacting with syntax). Notably, learners’ difficulties may often be observed only in online tasks because integrating grammatical and discourse information in real-time processing requires more cognitive resources.

The largest number of studies focusing on L2 processing of different word orders and relying on the Interface Hypothesis were conducted on Spanish (Lozano, 2006, 2014; Lozano and Mendikoetxea, 2008, 2010; Dominguez and Arche, 2014). In online experiments, even advanced L2 learners were shown to have some vestigial difficulties with SV/*VS* orders. At the same time, corpus studies suggest that they understand the syntax-discourse aspects of *VS* structures, although they may have some problems with the grammatical representation of non-subject preverbal XPs in such sentences.

Another formal Second Language Acquisition (SLA) theory that may be relevant for our study is the Bottleneck Hypothesis (Slabakova, 2014). In the generative framework, syntax relies on universal principles, while morphology is highly idiosyncratic and language-specific. Accordingly, the Bottleneck Hypothesis predicts that mastering syntax is much easier than mastering morphology, which is the primary source of problems for L2 learners.

Successfully processing different word orders in Russian definitely depends on the knowledge of morphology, most notably, case morphology. And we know that L2 learners of Russian have problems with it both in production and in comprehension until the most advanced levels (Rubinstein, 1995a, 1995b; Cherepovskaia et al., 2021, 2022). However, we must admit that based on the very few existing studies, so far it is impossible to tell whether L2 problems with non-canonical word orders in Russian are syntactic or morphological in nature and to what extent.

A general problem that is discussed in many functional approaches to SLA is the role of L1: it was confirmed to affect even advanced L2 learners, especially in the domain of discourse (Rutherford, 1983; Green et al., 2000; Han, 2000; Jung, 2004). Many studies of word order focus on cross-linguistic differences in the domain of verb subcategorization: which arguments are encoded as subjects or objects, how often a particular verb is used as transitive or intransitive (e.g., Frenck-Mestre and Pynte, 1997; Witzel et al., 2012).

Russian can provide a lot of interesting material to test the hypotheses outlined above and to establish the relative importance of different factors. However, this was done in only one study so far, which is also the only study assessing context effects on the L2 processing of different word orders. Laleko (2022) analyzed the role of information structure and predicate-argument structure in the processing of canonical and non-canonical orders for three groups of participants: native speakers, heritage speakers (low and high proficiency) and adult learners of Russian.

The study involved assessing the acceptability of SV (O) and (O) *VS* sentences in different contexts, i.e., unlike most studies discussed above, it did not use online measures. Three types of predicates were used: transitive, unergative and unaccusative verbs [for unaccusative verbs, *VS* is the neutral word order, see also (Slioussar, 2011b)]. Contexts were such that target sentences either had a broad focus (all information was new), as in (4a), or a narrow subject focus, as in (4b). After each context sentence, two target sentences with different word orders were presented, as in (4c-d), and participants were asked to rate both of them on a 1 to 5 scale.

(4) a. *Čto slučilos’?*

‘What happened?’

b. *Kto počinil velosiped?*

‘Who fixed the bicycle?’

c. *Papa počinil velosiped.* (SVO).

dad_NOM.SG_ fixed bicycle_ACC.SG_.

d. *Velosiped počinil papa.* (OVS).

bicycle_ACC.SG_ fixed dad_NOM.SG_.

Heritage and L2 speakers gave (O) *VS* structures lower ratings than native speakers. With SV and *VS* orders, information structure did not play a role for non-native speakers, but heritage speakers in the higher proficiency group were sensitive to the distinction between unaccusative and unergative verbs, like native speakers. With transitive verbs, higher proficiency heritage speakers demonstrated a native-like contrast in their ratings of OVS sentences with broad and narrow focus. Presumably, a given object may be a stronger trigger to use a non-canonical order than given information associated with the verb.

### 1.4 The present study

The goal of the present study was to compare L1 and L2 online and offline processing of different word orders in Russian. Our L2 participants were speakers of Mandarin Chinese. We chose SVO and OVS orders to have a canonical order and a non-canonical order with well-known information-structural properties and an inverted order of arguments, which is not characteristic for Chinese. Moreover, it was examined in several previous studies. In Experiment 1, target sentences were presented in isolation, while in Experiment 2, we used one-sentence contexts introducing one NP mentioned in the target sentence, like in several previous L1 studies (Kaiser and Trueswell, 2004; Slioussar, 2011a; Gattei et al., 2021).

Contexts introducing the first NP in the target sentence created a ‘given – new’ word order in it, which is characteristic for Russian, Chinese and many other languages with flexible word order (and, to a certain extent, to narrative texts universally). They can be viewed as appropriate. Contexts introducing the second NP created a ‘new – given’ word order in the target sentence and violated the information-structural requirements of OVS sentences (as we explained in section 1.1, SVO sentences are more flexible in this respect). They can be viewed as inappropriate. We aimed to find out how the word order factor and the context factor interact in L2 processing compared to L1 processing — a question that has been addressed in very few previous studies (and none of them compared appropriate and inappropriate contexts). This question was addressed in Experiment 2, while Experiment 1 examining the word order factor without the context factor can be seen as ancillary.

In both experiments, we measured word-by-word reading times to investigate online processing. After every sentence, we asked questions revealing whether readers interpreted it correctly, i.e., understood its predicate-argument structure. Finally, we also asked native speakers to evaluate how naturally target sentences sound on a 1 to 5 scale, tapping into their offline sensitivity to contextual requirements. Unlike Laleko (2022), we did not use this task with L2 participants (in her study, they were not sensitive to information-structural requirements of different word orders, only advanced heritage speakers were).

## 2 Experiment 1

In this experiment, our goal was to compare how native Russian speakers and Chinese learners process Russian sentences with different word orders (canonical SVO and inverted OVS) out of context and how native speakers evaluate them.

### 2.1 Participants

Two groups volunteered to take part in the study. The L1 group included 40 native Russian speakers (31 females) aged 18–43 (mean age 28.8). The L2 group consisted of 39 speakers of Mandarin Chinese (24 females) aged 18–35 (mean age 22.0). The experiment was carried out in accordance with the Declaration of Helsinki and existing Russian and international regulations concerning ethics in research. All participants provided informed consent. They received no financial reward for their participation.

All Chinese participants were students at Saint Petersburg State University in Russia and at the Belarusian State University in Belarus.^4^ They studied Russian at the Language testing center and at the Faculty of Philology of Saint Petersburg State University, and at the preparatory department of Belarusian State University. In total, 17 students were involved in different preparatory programs, 14 were in their first or second year of undergraduate studies, and 8 were in their third year. Twenty-three students had been living in Russia or Belarus for less than 1 year, 16 — for less than 2 years. When asked about their proficiency level in the Russian language, 19 people indicated the basic level (A2), 20 people — the lower intermediate, or the first certification level (B1). When asked about their proficiency in other foreign languages, all participants mentioned that they had studied English. Having more participants would be optimal, but the L2 groups we had access to (with a certain L1, a certain proficiency level etc.) were limited, unfortunately.

### 2.2 Materials

We constructed 16 sets of target sentences. Every set included two sentences that were identical except for the word order (SVO or OVS). Examples are given in (5a-b). We avoided object experiencer psych verbs or other constructions in which non-canonical orders may be more frequent than the canonical one (these verbs were discussed in the section 1.2). Since all sentences in the experiment were presented to participants segment-by-segment, we indicate how they were divided into segments.

(5) а. *Russkij prepodavatel’ / slušaet / kitajskogo studenta/i smotrit v okno*.

Russian_NOM.SG_ teacher_NOM.SG_ / listens / Chinese_ACC.SG_ student_ACC.SG_ / and looks in window.

b. *Kitajskogo studenta / slušaet / russkij prepodavatel’ /i smotrit v okno*.

Chinese_ACC.SG_ student_ACC.SG_ / listens / Russian_NOM.SG_ teacher_NOM.SG_/ and looks in window.

‘A / the Russian teacher is listening to a / the Chinese student and looking out the window.’

Thus, each sentence consisted of the following four segments:

- a subject NP (an animate noun in nominative singular with a preposed adjective);
- an object NP (an animate noun in accusative singular with a preposed adjective);
- a transitive verb in the present or past tense;
- the final segment (a second coordinated VP, a PP depending on the first verb etc.).

As we mentioned in the introduction, animacy may affect word order in the absence of information-structural differences. Therefore, subject and object NPs were balanced with respect to the animacy scale (both denoted either humans or animals). We chose only animate nouns to avoid forms with case syncretism (in most inanimate nouns, accusative forms coincide with nominative ones). The segments containing the object and the subject, which were crucial for our study, always consisted of two words. This was done to make reading time differences more pronounced. The final segment was introduced so that subject and object segments were not sentence-final.

We made sure that in all target sentences, it was impossible to guess grammatical roles of the NPs based on the semantics alone. For example, if (5a-b) are considered, both the teacher can look at the student and vice versa. Therefore, participants had to rely on case information to interpret these sentences correctly. To assess their interpretation accuracy, we constructed two questions for each target sentence set directed at the subject and at the object, like in (6a-b). All questions contained only a question word and a verb — we did not want to give our participants any further hints or to confuse them any further by adding any NPs.

(6) а. *Kto slušaet?*

who_NOM.SG_ listens.

‘Who is listening?’

b. *Kogo slušajut*?

whom_ACC.SG_ listen.

‘Who is being listened to?’^5^

Rather than giving participants a choice of two answers, as it is usually done, we provided them with a window to type in their answer. This made the task more difficult for the L2 group, but we wanted to avoid guessing. The instructions before the experiment specified that brief answers (only the noun) were acceptable. Every participant saw an equal number of target sentences in the two experimental conditions (SVO and OVS) with an equal number of subject and object questions.

Thus, every participant read 16 target sentences in one of the two conditions. We also constructed 10 filler sentences that were more syntactically diverse than target sentences to distract participants’ attention from the experimental manipulation. Two examples are given in (7a-b). The questions for the fillers were directed at the PPs with a temporal or locative meaning. Filler sentences and questions were the same for every participant.

(7) а. *K našemu deduške redko prixodjat raznye gosti.*

to our_DAT.SG_ grandfather_DAT.SG_ rarely come various_NOM.PL_ guests_NOM.PL_.

b. *Bednyj xudožnik uexal iz Peterburga v pjatnicu večerom.*

poor_NOM.SG_ artist_NOM.SG_ left from Petersburg_GEN.SG_ on Friday_ACC.SG_ at-night.

When constructing target and filler sentences, we selected vocabulary and grammatical features in accordance with the lexical minima and state standards for Russian as a foreign language. We made sure that they did not exceed the basic level according to the Russian State Testing System. Additionally, all sentences were checked on the online platform *Textometr.*^6^ They were generally assessed as being at the A1 level (elementary), and the A2 lexical list covered 87% of the vocabulary.

### 2.3 Procedure

The experiment was run on the web-based platform PCIbex.farm. Data were collected in the presence of the experimenter or the Russian teacher of the L2 participants. We created two versions of the experiment for the L1 and L2 groups. In both groups, we measured sentence reading times and question answering accuracy. After that, the L1 group received a second task (evaluating target and filler sentences on a scale), while the L2 group was asked to fill in a questionnaire (about their native and foreign languages, and about their Russian studies in particular). For the L2 group, the experimental instructions and the questionnaire were translated into Chinese and checked by a native Chinese speaker.

To measure reading times, we used the moving window self-paced reading task (Just et al., 1982). Each trial began with a screen presenting a sentence, in which the words were masked by dashes, while spaces and punctuation remained intact. Each time the participant pressed the space bar, a segment was revealed, the previous segment was re-masked, and RTs were measured. After each sentence, a question and a window to type in the answer appeared. Participants were instructed to read at their natural pace. Two practice items were presented before the beginning of the experiment (in particular, we made sure that L2 readers understood the questions by giving them feedback).

In the second part of the experiment, L1 participants were asked to evaluate a number of sentences on the 1 to 5 scale, where 5 indicated a sentence that sounded fully natural in Russian, and 1 indicated a sentence that sounded completely unnatural. We included all target sentences from the first part in this task, as well as four filler sentences in which we modified the word order in a way that is not characteristic for Russian (although grammatical). We were interested to find out whether L1 participants subjectively perceive non-canonical word orders as sounding less natural than canonical ones in zero context. Sentences were shown on the screen one by one (unmasked). Before the main session, two practice items were presented.

### 2.4 Analysis

We analyzed participants’ reading times, question answering accuracy and sentence ratings (in the L1 group). We did not analyze response times (these data were too noisy because our participants had to type their answers). Data from the two groups were analyzed separately.

During the preliminary data processing in the L1 group, RTs that exceeded a threshold of 2.5 standard deviations, by segment and by condition, were excluded as outliers (Ratcliff, 1993). In total, this led to the exclusion of 4.8% of the data. There was no filtering based on accuracy since all participants performed well, providing over 86% correct responses.

In the L2 group, the task appeared to be too difficult for many participants, as we could judge from their low accuracy. To have an exclusion criterion independent from our experimental manipulations, we discarded data from participants who scored below 60% correct on questions to filler sentences. As a result, data from 26 out of 39 participants were included in the final analysis. Subsequently, 5.0% of the RTs were excluded because they exceeded a threshold of 2.5 standard deviations, by segment and by condition.

The statistical analysis was done in the *R* programming environment.^7^ We modeled RT data with a mixed-effects regression using the *lmer* function from the *lme4* package, accuracy data with a mixed-effects logistic regression using the *glmer* function from the *lme4* package, and sentence rating data with a mixed-effects ordinal regression using the *glmer* function from the *lme4* package (Bates et al., 2015). To obtain the *p* values from the *t* values given by the model, we used the *lmerTest* package (Kuznetsova et al., 2015). Random intercepts and random slopes by a participant and by an item were included in the models.

In the analysis of sentence ratings, word order (SVO or OVS) was the only fixed effect. In the analysis of RTs, we added segment length. Some NPs and verbs that we used were longer than the others, so it was significant in most comparisons, as expected. But this variation could have been covered by random effects, so this factor was not interesting to us *per se*. The reason to include it was that some accusative singular forms of nouns and adjectives are one letter longer than nominative forms, and we wanted to make sure that if there are any differences between the two experimental conditions, they cannot be reduced to that. In the analysis of answering accuracy, we used the word order factor and two factors capturing to which NP the question was directed: to the subject or the object (NP role) and to the first or the third segment (NP position). A preliminary examination of the data from the L2 group suggested that the later factor may be important, and the subsequent statistical analysis confirmed that.

### 2.5 Results

#### 2.5.1 L1 group: reading times

**Figure 1:**
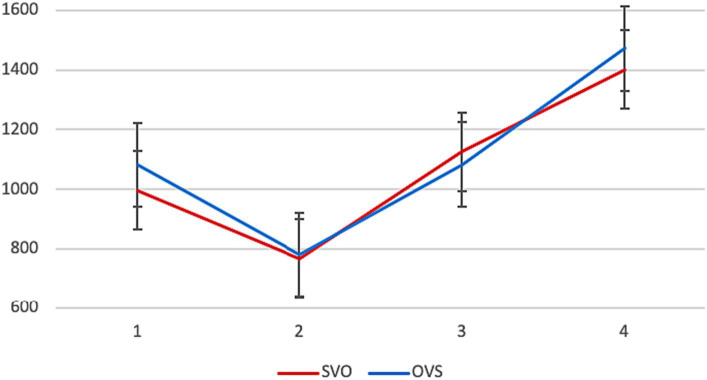
Experiment 1, L1 group: average reading times per segment (in ms) in different conditions.

Average reading times in different conditions are presented in Figure 1. No significant differences between the two word orders were found.^8^

#### 2.5.2 L1 group: question answering accuracy

The average proportion of correct answers by condition is presented in Table 1. Out of three factors we analyzed, the word order proved to be significant. Overall, L1 participants performed very well, but made more errors with OVS sentences (*β* = −0.78, SE = 0.31, *p* = 0.013).

**Table 1:** Experiment 1, L1 group: the proportion of correct answers in different conditions.

Word order	NP role	NP position	Correct answers
SVO	S	1	93%
SVO	O	3	92%
OVS	O	1	87%
OVS	S	3	86%

#### 2.5.3 L1 group: sentence ratings

Average ratings of SVO and OVS sentences are presented in Table 2. The SVO order was rated significantly higher than the OVS one (*β* = −2.65, SE = 0.75, *p* < 0.001).

**Table 2:** Experiment 1, L1 group: average ratings of sentences in different conditions.

Word order	Rating
SVO	4.48
OVS	3.05

#### 2.5.4 L2 group: reading times

**Figure 2:**
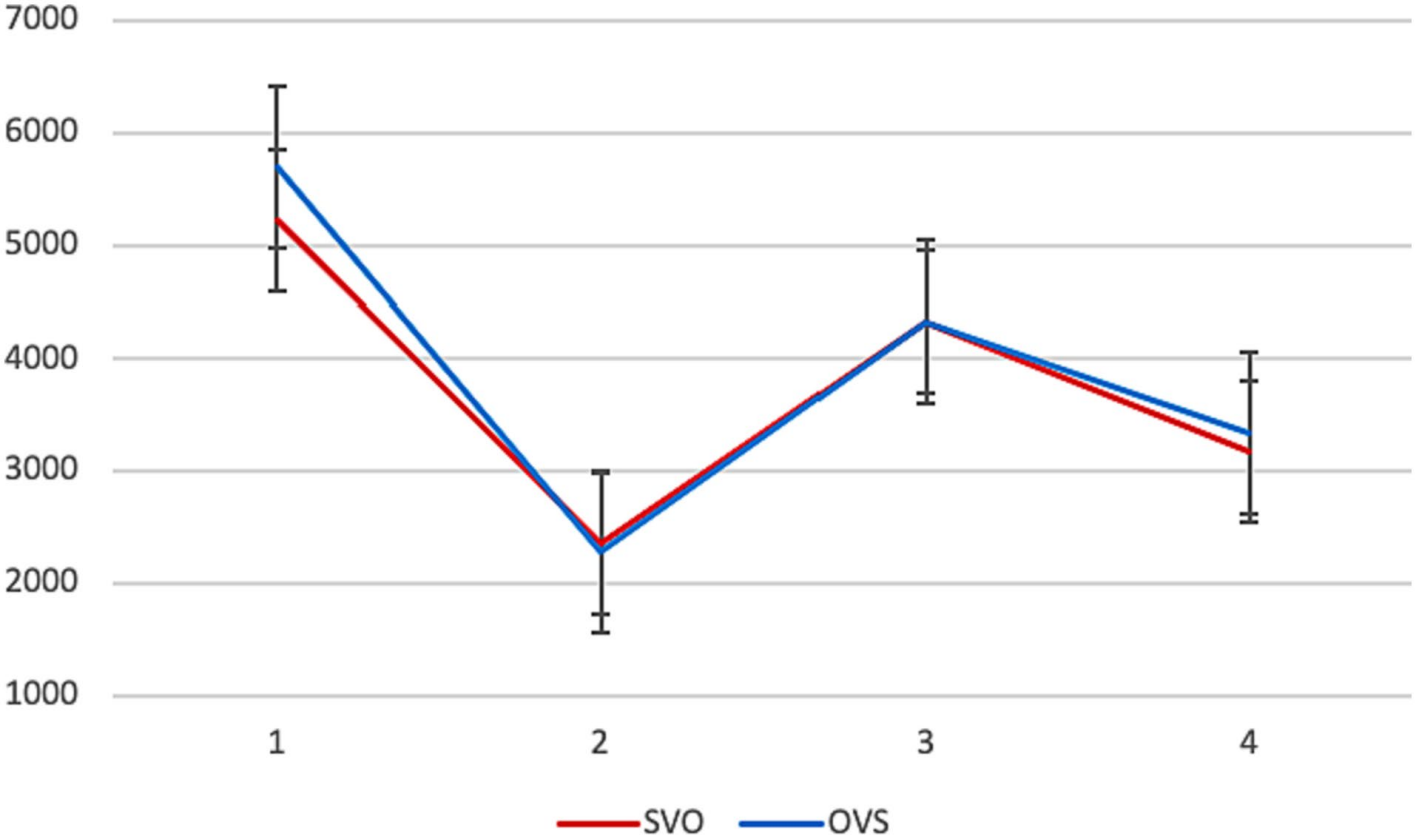
Experiment 1, L2 group: average reading times per segment (in ms) in different conditions.

Average reading times are presented in Figure 2. Significant differences between the two conditions were found in the first segment. The first NP is read faster when it is the subject (in SVO) than when it is the object (in OVS) (*β* = 726.80, SE = 290.77, *p* = 0.013).^9^

#### 2.5.5 L2 group: question answering accuracy

The average proportion of correct responses by condition is given in Table 3. Two factors were significant: the word order and the NP position, while the NP role was not. It was easier for L2 participants to answer questions about SVO sentences (*β* = −0.56, SE = 0.21, *p* = 0.011) and about the first NP in the sentence (*β* = −0.92, SE = 0.22, *p* < 0.001). Maybe, this NP was better memorized. Another possibility was suggested by an anonymous reviewer. In Chinese, *wh*-phrases do not have a designated position in the beginning of the sentence, i.e., they stay *in situ*, so subject *wh*-phrases are preverbal, like NP subjects, while object *wh*-phrases follow the verb, like NP objects. Maybe, our participants sometimes treated all *wh*-words and all preverbal NPs as subjects, which gave them a chance to respond correctly when neither of these assumptions was correct.

**Table 3:** Experiment 1, L2 group: the proportion of correct answers in different conditions.

Word order	NP role	NP position	Correct answers
SVO	S	1	74%
SVO	O	3	46%
OVS	O	1	55%
OVS	S	3	43%

### 2.6 Discussion

The goal of this experiment was to examine how isolated sentences with different word orders are processed by native speakers of Russian and by learners of Russian as a foreign language. In online processing, the OVS order did not pose any significant difficulties for L1 readers, but interpretation accuracy for OVS sentences was slightly lower. This is expected given that sentences with non-canonical orders are widespread in Russian, although the canonical order is still by far the most frequent. However, word order alternations are regulated by information structure, so non-canonical orders have certain contextual requirements and are not used in isolation. We demonstrated that native speakers are sensitive to that when they evaluate how natural sentences with different orders sound to them.

The picture is different for L2 readers. In OVS sentences, they slow down on the first segment, when it becomes clear that they are dealing with a non-canonical word order. Their interpretation accuracy shows that online difficulties often result in the ultimate failure to construct a correct interpretation, in particular, to understand the predicate-argument structure of the sentence. The fact that L2 readers answer questions about the first NP in the sentence more accurately also stresses that processing several arguments and understanding their semantic roles is difficult for them. Difficulties associated with understanding *wh-*questions may aggravate the situation. To tease apart these factors, one may turn to a different experimental design, in which participants are asked to choose a picture that corresponds to a sentence rather than to answer *wh*-questions.

At the same time, let us note that we do not see any significant differences associated with case *per se* (it could be the case that accusative NPs had longer RTs in any position or triggered more interpretation errors). L2 readers resemble L1 readers in this respect, but the similarity may be deceiving. For L1 readers, processing case information is too easy to produce any noticeable effects. For our L2 group, it may be too difficult: maybe, we do not see any effects because they usually fail to do so, which is eventually reflected in their low question-answering accuracy. Further studies with more advanced L2 participants are necessary to find a definitive answer.

Finally, let us note the following difference between L1 and L2 groups. L2 participants take the longest to read the first segment, while RTs for the final segment, which reflect late stages of syntactic processing, are relatively short compared both to the first and the third segment. In contrast, L1 readers process NPs in the first and the third segments relatively fast and slow down on the last segment — presumably, to complete the syntactic representation of the sentence. Judging by their low accuracy, L2 participants often skip this step, being overloaded with syntactic processing, and, consequently, fail to arrive at the correct interpretation of the sentence.

## 3 Experiment 2

In this experiment, we aimed to compare how native Russian speakers and Chinese learners of Russian process sentences with different word orders (SVO and OVS) in the contexts satisfying or not satisfying their information-structural requirements. We also tested how native speakers evaluate them.

### 3.1 Participants

Like in Experiment 1, there were two groups of participants. The L1 group included 51 native Russian speakers (38 females) aged 17–47 (mean age 27.0). The L2 group consisted of 44 speakers of Mandarin Chinese (27 females) aged 18–25 (mean age 21.4). The experiment was carried out in accordance with the Declaration of Helsinki and existing Russian and international regulations concerning ethics in research. All participants provided informed consent and volunteered to participate without any financial reward.

All Chinese participants studied Russian at the Language testing center or at the Faculty of Philology of Saint Petersburg State University. In total, 5 students were involved in preparatory programs, 14 were in their first or second year of undergraduate studies, and 25 were in their third year. Sixteen students had been living in Russia for less than 1 year, 15 — for less than 2 years, 13 — for less than 3 years. When asked about their proficiency level in the Russian language, 9 students indicated the basic level (A2), and 35 people — the lower intermediate, or the first certification level (B1). Additionally, they all mentioned that they also studied English as a foreign language.

### 3.2 Materials

We took 16 target sentence sets from Experiment 1 (including questions) and constructed two one-sentence contexts for them. Context sentences always mentioned the subject or object from the target sentence. Examples are given in (8a-b) (in (9a-b), we repeat examples of target sentences given in (5a-b) above).

(8) а. *Russkij prepodavatel’ / provodit / zanjatie v auditorii*.

Russian_NOM.SG_ teacher_NOM.SG_ / conducts / lesson in classroom.

‘A Russian teacher is conducting a lesson in the classroom.’

b. *Kitajskij student / prišel / na zanjatie v auditoriyu*.

Chinese_NOM.SG_ student_NOM.SG_ / came / to lesson in classroom.

‘A Chinese student came to a lesson in the classroom.’

(9) а. *Russkij prepodavatel’ / slušaet / kitajskogo studenta / i smotrit v okno*.

Russian_NOM.SG_ teacher_NOM.SG_ / listens / Chinese_ACC.SG_ student_ACC.SG_ / and looks in window.

b. *Kitajskogo studenta / slušaet / russkij prepodavatel’ / i smotrit v okno*.

Chinese_ACC.SG_ student_ACC.SG_ / listens / Russian_NOM.SG_ teacher_NOM.SG_ / and looks in window.

‘The Russian teacher is listening to the Chinese student and looking out the window.’

If we present (9a) after (8a) and (9b) after (8b), target sentences will start with a given NP followed by a new one (we will term this *G-N contexts*). This is characteristic for languages with a free word order, including Russian, so we can consider G-N contexts appropriate for the respective target sentences, or satisfying their information-structural requirements. If we present (9a) after (8b) and (9b) after (8a), target sentences will start with a new NP followed by a given one (*N-G contexts*). N-G contexts are infrequent in Russian and can be found only in special constructions like focus fronting. In our case, no focus fronting can be expected, so these contexts can be considered inappropriate, or not satisfying the information-structural requirements of target sentences. As we noted in the introduction, the canonical SVO order is compatible with a wider range of contexts, while other orders, like OVS, have much stricter context requirements. In this study, we aim to find out whether participants are sensitive to N-G contexts in general and to the contextual requirements of different orders.

Context sentences satisfied the same requirements for vocabulary and grammar as target sentences did. The character from the target sentence was mentioned at the beginning or in the middle of the context sentence to give readers some time to accommodate this information. We used the same NP as in the target sentence (to leave no room for confusion) in the nominative singular form. This is a potential limitation of our study that can be addressed in further research: for L2 participants, it may be easier to read target sentences in which the given NP is in the same case as in the context sentence. Pairs of context sentences could be different in the beginning, but the end was always the same to avoid any effects in the following target sentence. We made sure that context sentences do not provide any hints on the distribution of grammatical roles in target sentences.

We also took 10 filler sentences with questions from Experiment 1 and created context sentences for them. These context sentences did not vary and could be considered appropriate (G-N). In the second part of the experiment, in which L1 participants rated sentences, four filler sentences with a modified word order were used, like in Experiment 1. Since the word order changed, the context became N-G (inappropriate).

### 3.3 Procedure

The procedure was the same as in the Experiment 1.

### 3.4 Analysis

Like in Experiment 1, we analyzed participants’ reading times in target sentences (context sentence data were not included in the analysis), question answering accuracy and sentence ratings (in the L1 group). Data from the two groups were analyzed separately. During the preliminary data processing, we excluded 12 out of 44 L2 participants who gave less than 60% correct answers to the questions to filler sentences. In the L1 group, all participants answered more than 85% questions correctly. Then RTs that exceeded a threshold of 2.5 standard deviations, by segment and by condition, were removed (3.7% of the data in the L1 group, 5.4% in the L2 group).

The statistical analysis was the same as in Experiment 1. In the analysis of RTs and ratings, two fixed effects were included: word order (SVO or OVS) and context (G-N or N-G). In the discussion section, we will come back to the question which context effects can be explained by its global and local properties (i.e., by its general (in)appropriateness or by givenness/newness of certain NPs).

As for answering accuracy, we should note that due to a technical issue, there was a problem with the design: questions related to the subject were always asked after sentences in a G-N context, while questions related to the object were asked after sentences in a N-G context. Thus, the NP role factor (subject or object) was coupled with the context factor, and the word order factor was coupled with NP givenness. The NP position factor remained independent. Thus, we can interpret the obtained results only with significant limitations, but will nevertheless propose an interpretation in the discussion section.

### 3.5 Results

#### 3.5.1 L1 group: reading times

**Figure 3:**
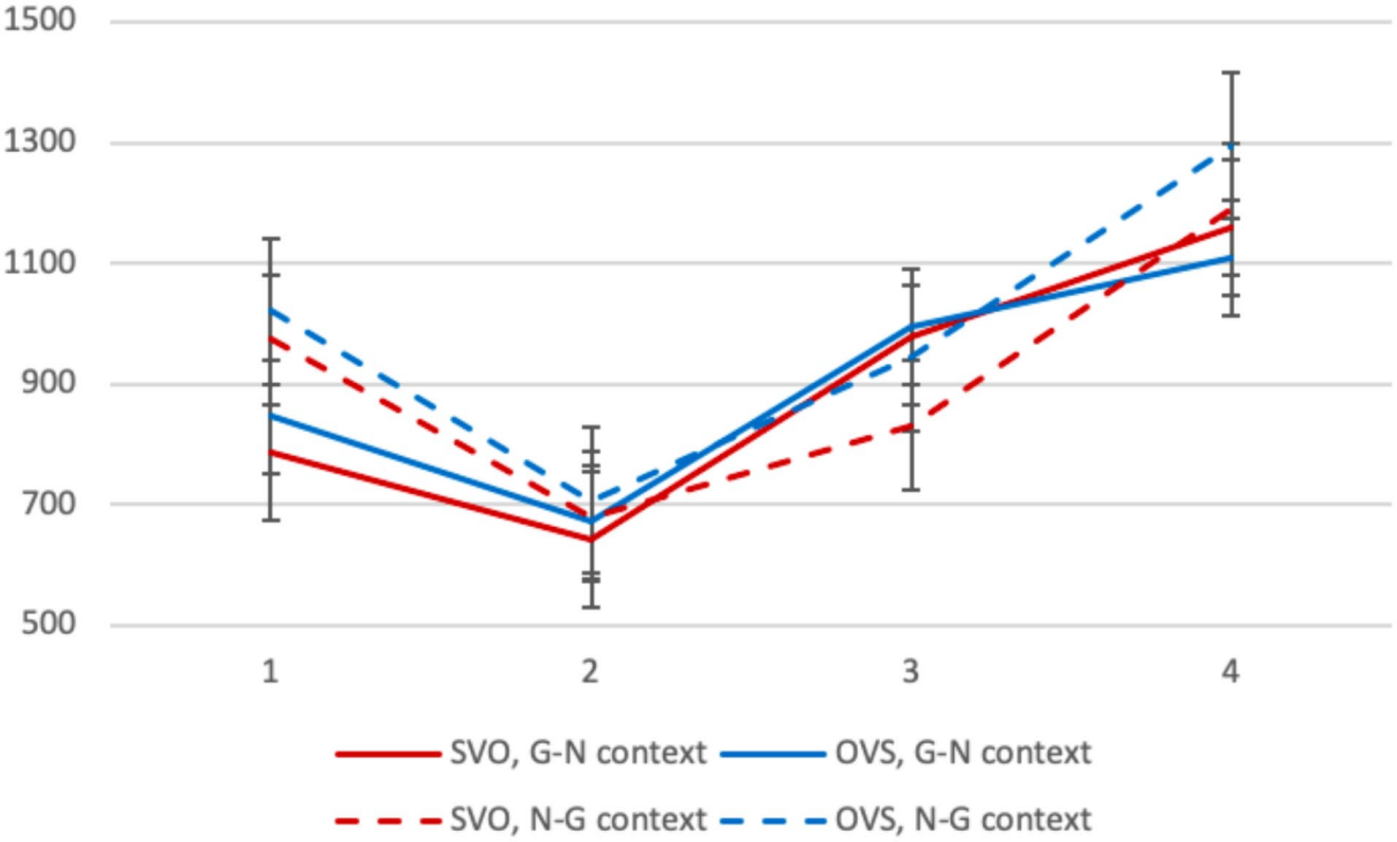
Experiment 2, L1 group: average reading times per segment (in ms) in different conditions.

Average reading times in different conditions are presented in Figure 3. On the first segment, the context factor was significant: sentences in the G-N context were read faster (*β* = 181.79, SE = 30.61, *p* < 0.001). Of course, this effect may be explained, at least partially, by the fact that given NPs are read faster than new ones (especially given the fact that they were literally repeated). There were no significant differences between conditions on the second segment.^10^

On the third segment, the context factor was significant, and this was definitely due to NP givenness: given NPs in N-G contexts were read faster (*β* = −145.70, SE = 34.53, *p* < 0.001). The interaction between the two factors reached significance as well: OVS sentences in the N-G context were read slower than SVO ones (*β* = 106.49, SE = 48.94, *p* = 0.030). This cannot be explained by the local properties of NPs and is most probably due to the fact that OVS sentences have much stricter context requirements, and native speakers are sensitive to that. On the final segment, there was a significant interaction between the two factors, similar to that observed for the third segment (*β* = 168.85, SE = 83.20, *p* = 0.043). The effect of context was visible for OVS sentences, but not for SVO ones.

#### 3.5.2 L1 group: question answering accuracy

The average proportion of correct answers by condition is presented in Table 4. L1 participants performed very well, and no differences reached significance, although certain tendencies can be seen.

**Table 4:** Experiment 2, L1 group: the proportion of correct answers in different conditions.

Context	Word order	NP role	NP position	NP givenness	Correct answers
G-N	SVO	S	1	given	91%
N-G	SVO	O	3	given	88%
G-N	OVS	S	3	new	88%
N-G	OVS	O	1	new	86%

#### 3.5.3 L1 group: sentence ratings

Average ratings of sentences in different conditions are presented in Table 5. G-N contexts were rated significantly higher (*β* = −1.62, SE = 0.21, *p* < 0.001). The interaction was also significant: like with the RT data, the effect of context was more pronounced for OVS sentences (*β* = −1.54, SE = 0.29, *p* < 0.001).

**Table 5:** Experiment 2, L1 group: average ratings of sentences in different conditions.

Context	Word order	Rating
G-N	SVO	4.44
N-G	SVO	3.84
G-N	OVS	4.41
N-G	OVS	3.06

#### 3.5.4 L2 group: reading times

**Figure 4:**
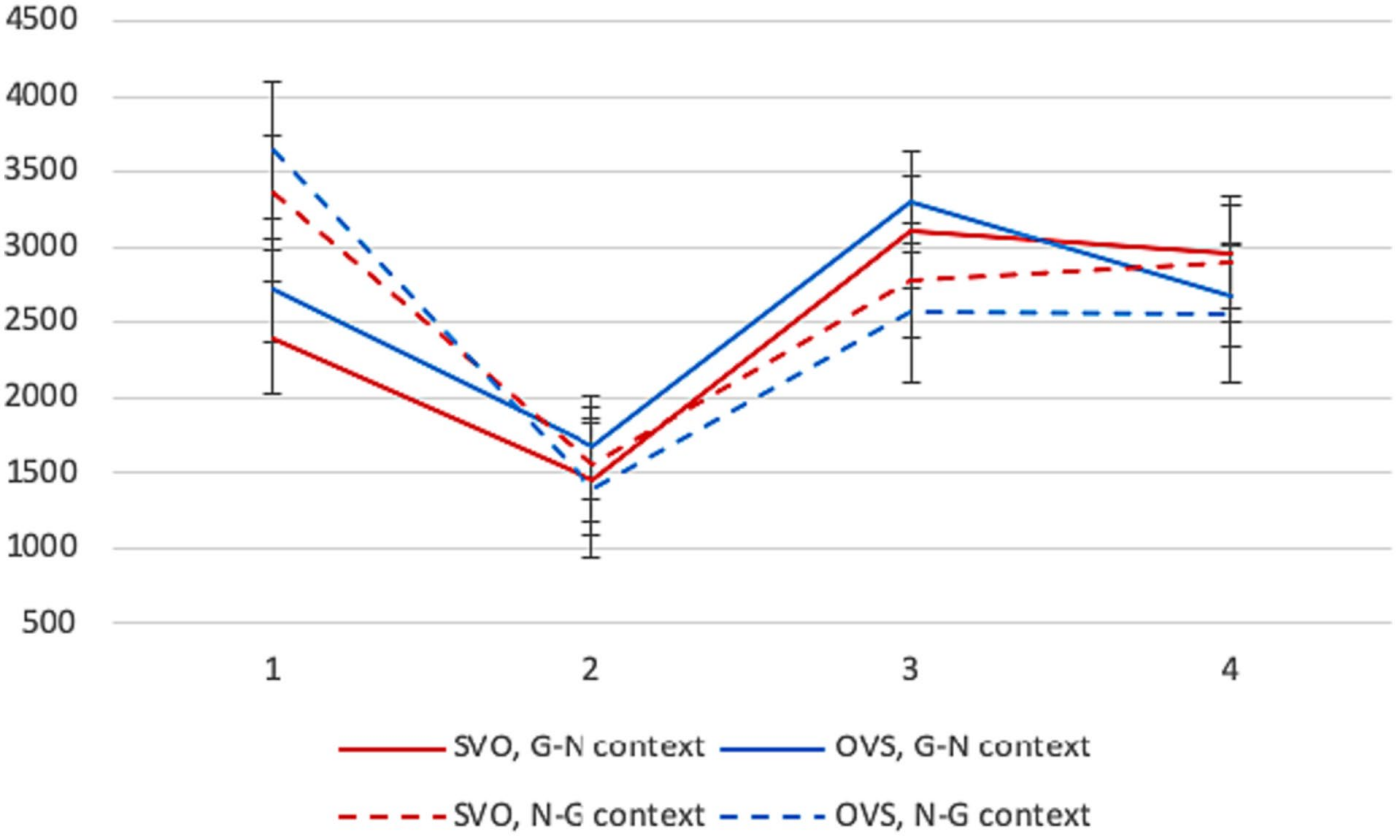
Experiment 2, L2 group: average reading times per segment (in ms) in different conditions.

Average reading times in different conditions are presented in Figure 4. The context factor was highly significant on the first segment, like for L1 participants. Given NPs in the G-N context were read much faster (*β* = 945.92, SE = 225.42, *p* < 0.001). The word order factor reached significance as well: SVO was easier (*β* = 389.94, SE = 230.35, *p* = 0.032).^11^

There were no significant differences between conditions on the second segment. On the third segment, the context factor was significant: given NPs in N-G contexts were read faster (*β* = −572.29, SE = 208.41, *p* = 0.006). This result was similar to the L1 group, but other patterns were not. The interaction between the context and word order factors reached significance: the effect of givenness was more pronounced for OVS sentences (*β* = −359.15, SE = 292.75, *p* = 0.041). Notably, L1 participants read OVS sentences in N-G contexts more slowly than SVO sentences, being sensitive to their stricter context requirements. We do not see this sensitivity in L2 readers who show the opposite pattern. No differences between conditions reached significance on the final segment.

#### 3.5.5 L2 group: question answering accuracy

The average proportion of correct answers by condition is presented in Table 6 and summarized in Table 7. As we noted above, due to a technical issue, questions related to the subject were always asked after G-N sentences, while questions related to the object — after N-G sentences. Therefore, some factors were coupled, and we should try to tease them apart when interpreting the results.

**Table 6:** Experiment 2, L2 group: the proportion of correct answers in different conditions.

Context	Word order	NP role	NP position	NP givenness	Correct answers
G-N	SVO	S	1	given	80%
N-G	SVO	O	3	given	62%
G-N	OVS	S	3	new	37%
N-G	OVS	O	1	new	46%

**Table 7:** Experiment 2, L2 group: the proportion of correct answers depending on different factors.

Word order + NP givenness		Context / NP role		NP position	
SVO + given	OVS + new	G-N + S	N-G + O	1	3
71%	42%	59%	54%	63%	50%

Two groups of factors significantly affected the results: the word order/givenness (*β* = −1.37, SE = 0.20, *p* < 0.001) and the NP position (*β* = −0.70, SE = 0.20, *p* < 0.001). In case of word order and givenness, both factors probably influenced the results working in the same direction and resulting in the most pronounced effect (the former was significant in Experiment 1 and for RT data in the current experiment, the latter was the main factor affecting RTs). The NP position factor that played a role in Experiment 1 remained significant, as expected.

The context type and NP role factors, which did not reach significance, could not cancel each other out because they were supposed to work in the same direction. The NP role did not affect answering accuracy in Experiment 1 or RTs in both experiments. The effects of context on RTs in the L2 group are mostly local. L2 participants find given NPs easier to read (which was captured by the NP givenness factor in the current analysis), but are not sensitive to the global (in)appropriateness of the context.

### 3.6 Discussion

The goal of the second experiment was to compare how sentences with different word orders are processed by L1 and L2 participants in different contexts: G-N and N-G. Similarly to Experiment 1, the word order factor significantly affected RTs and question-answering accuracy only in the L2 group. For L1 participants, processing non-canonical orders was not particularly difficult.

The context factor played a major role both for L1 and for L2 participants. As we noted above, its effects can be explained locally (by the givenness of particular NPs) or globally (by the fact that G-N contexts are characteristic for Russian and can be seen as appropriate, while N-G contexts are not). A slowdown associated with it was much larger on the first segment (on new NPs in the N-G context) than on the third segment (on new NPs in the G-N context) in both groups. We can conclude that for all readers, both local and global aspects are important, although local ones play a larger role.^12^ Sentence ratings in the L1 group can be affected only by the global (in)appropriateness of the context, and we can see that this factor was significant.

However, while the global RT picture is similar for L1 and L2 participants, there are also some principled differences. For L1 readers, the effect of the inappropriate N-G context is more pronounced for OVS orders. This is evident not only in RTs (given subjects following new objects are read almost as slowly as new NPs), but also in sentence ratings. This can be explained by more strict context requirements for non-canonical orders. L2 readers do not exhibit a similar subtle sensitivity to context.

Finally, let us note that NP position affected accuracy in the L2 group, like in Experiment 1. It was easier to answer questions about the first NP in the sentence. Given that the overall accuracy was low, this supports the conclusion that L2 readers have problems with processing several arguments and understanding their semantic roles. Problems with understanding *wh*-questions could also contribute to this.

## 4 Conclusion

In this study, we compared how native speakers of Russian and speakers of Mandarin Chinese learning Russian as a foreign language process Russian sentences with different word orders in isolation (Experiment 1) and in context (Experiment 2). We chose SVO and OVS sentences for the comparison to have a canonical order and a non-canonical order with well-known information-structural properties and an inverted order of arguments, which is not characteristic for Chinese. One-sentence contexts introduced one NP mentioned in the target sentence, either the first or the second. Thus, in the former case, given information preceded new information in the target sentence, which is characteristic for Russian and many other languages, while in the latter case, the opposite was true. We used different measures to capture online and offline effects: word-by-word reading times, question-answering accuracy and sentence rating on a 1 to 5 scale (for L1 participants).

In both experiments, RTs and question-answering accuracy data showed that non-canonical orders were difficult for L2 participants, but not for L1 participants (for them, the effects of this factor were small or absent altogether). However, L1 participants gave non-canonical orders lower ratings in isolation, presumably because in naturally occurring texts, they are used only in particular contexts. It would be interesting to find out to what extent these difficulties are universal for L2 processing, or native speakers of other languages in which subject-object inversion is possible, like in Russian, would not experience them. Further research may also focus on other non-canonical word orders. For example, would SOV be more difficult than SVO for L2 readers, or only changing the relative order of arguments creates substantial problems? How would SV(XP) vs. (XP)*VS* orders with intransitive verbs, like the ones examined by Laleko (2022), be processed?

As for the context factor in Experiment 2, some effects are universal for L1 and L2 processing: all participants read given NPs faster than new ones and preferred sentences with a ‘given – new’ word order. The latter may reflect the universal principles of narrative coherence — then L2 readers do not need to acquire this knowledge, they only need to apply it to a new language. However, unlike native speakers, they are not sensitive to more subtle contextual requirements of different word orders, in particular, to the fact that the canonical word order is acceptable in a much wider range of contexts, while non-canonical orders heavily depend on the appropriate context to sound natural. These results are interesting to compare with those by Laleko (2022) who found that L2 learners are not sensitive to information-structural requirements when asked to evaluate SV(O) and (O)*VS* sentences. Thus, although it might be easier for them to process ‘given – new’ orders, this does not necessarily crystallize into knowledge how different orders should be used.

## Data availability statement

The datasets presented in this study can be found in online repositories. The names of the repository/repositories and accession number(s) can be found at: https://osf.io/9eust/?view_only=d5205d3a3b7644e595e4910470e4f095.

## Ethics statement

Ethical approval was not required for the studies involving humans because The local Russian legislation does not require ethics committee approval for behavioral studies with adult non-vulnerable participants IF they provide written informed consent and IF the study is carried out in accordance with the Declaration of Helsinki and existing Russian and international regulations concerning ethics in research. The studies were conducted in accordance with the local legislation and institutional requirements. The participants provided their written informed consent to participate in this study.

## Author contributions

NS: Conceptualization, Investigation, Project administration, Writing – original draft. MH: Data curation, Investigation, Writing – original draft.

## Footnotes

^1^Here and below, we discuss affirmative sentences, questions may be different in this respect.

^2^Sometimes this is not possible due to case syncretism (for example, accusative forms coincide with nominative ones in most inanimate nouns), and the reader has to rely on context and on the primacy of the canonical word order. We avoided such cases in our study.

^3^This group is not very numerous, so it does not undermine the overall prevalence of the SVO order.

^4^The teaching at the Belarusian State University is in Russian, and the absolute majority of people in Minsk where it is located also speak Russian as their native language. The Belarusian language is more widespread in other places in Belarus.

^5^The verb is in the 3rd person plural form that can be used when the subject remains unspecified. It was important for us that in the question about the subject, the *wh*-word is in nominative, and in the question about the object, it is in accusative, and both questions do not contain any other NPs.

^6^ https://textometr.ru/

^7^ www.r-project.org

^8^The length factor was significant in the first, second and fourth segments (*β* = 31.72, SE = 9.89, *p* = 0.005; *β* = 23.89, SE = 7.96, *p* = 0.009; *β* = 47.41, SE = 9.67, *p* < 0.001).

^9^The length factor was significant in the second segment (*β* = 214.60, SE = 38.54, *p* < 0.001).

^10^The length factor was significant in the second, third and fourth segments (*β* = 9.46, SE = 4.30, *p* = 0.045; *β* = 28.51, SE = 5.76, *p* < 0.001; *β* = 34.15, SE = 4.05, *p* < 0.001).

^11^The length factor was significant in the second and fourth segments (*β* = 133.91, SE = 24.44, *p* < 0.001; *β* = 70.90, SE = 22.04, *p* = 0.006).

^12^Most probably, they also affected accuracy in the L2 group, but their effects cannot be teased apart from the word order effects (in the L1 group, no factor reached significance in this task).
